# Body mass index and physical activity in early childhood are associated with atopic sensitization, atopic dermatitis and asthma in later childhood

**DOI:** 10.1186/s13601-016-0124-9

**Published:** 2016-08-24

**Authors:** Kristine Kjer Byberg, Geir Egil Eide, Michele R. Forman, Pétur Benedikt Júlíusson, Knut Øymar

**Affiliations:** 1 Department of Pediatrics, Stavanger University Hospital, POB 8100, 4068 Stavanger, Norway; 2 Centre for Clinical Research, Haukeland University Hospital, Bergen, Norway; 3 Department of Global Public Health and Primary Care, University of Bergen, Bergen, Norway; 4 Department of Nutritional Sciences, School of Human Ecology, University of Texas at Austin, Austin, TX USA; 5 Department of Pediatrics, Haukeland University Hospital, Bergen, Norway; 6 Department of Clinical Science, University of Bergen, Bergen, Norway

**Keywords:** Allergic rhinitis, Anthropometry, Asthma, Atopic dermatitis, Child

## Abstract

**Background:**

The results of studies on the associations of childhood excessive weight/obesity and physical activity with atopic sensitization and atopic diseases are inconsistent. We studied the associations of anthropometry and physical activity in childhood with atopic sensitization and atopic diseases in late childhood.

**Methods:**

In a cohort study including cases exposed to preeclampsia during pregnancy and controls, anthropometry and physical activity were assessed at several ages in 617 children. Associations with atopic sensitization and atopic diseases in late childhood were analysed using multiple logistic regression.

**Results:**

Body mass index standard deviation score (BMI SDS) at 1 year and low physical activity at 3–6 years were positively associated with atopic sensitization at 12.8 years [adjusted odds ratio (OR) 1.22; 95 % confidence interval (1.00, 1.49) and OR 2.36; (1.15, 4.81), respectively]. Change in BMI SDS from 1 to 4 years, BMI SDS at 4 years, and high physical activity at 6–10 years were positively associated with atopic dermatitis by 10.8 years [OR 1.46; (1.11, 1.92); OR 1.32; (1.06, 1.65) and OR 1.94; (1.16, 3.24); respectively]. Low physical activity at 3–6 and 6–10 years were positively associated with asthma by 10.8 years [OR 3.61; (1.56, 8.36) and OR 2.52; (1.24, 5.12), respectively].

**Conclusions:**

BMI and physical activity in early childhood were associated with atopic sensitization, atopic dermatitis and asthma in later childhood. Larger cohorts with repeated measurements of both predictors and outcomes are required to further elucidate this issue.

*Trial registration* Our study was observational without any clinical intervention on the participants. Therefore, no trial registration number is available

**Electronic supplementary material:**

The online version of this article (doi:10.1186/s13601-016-0124-9) contains supplementary material, which is available to authorized users.

## Background

The prevalence of obesity, allergy and asthma has increased worldwide during the last decades [1, 2]. An association between obesity and asthma has been suggested both in early and late childhood [3], where obesity precedes asthma in prospective studies [4]; the association between obesity and allergy is inconsistent [5].

High birth weight or body mass index (BMI; kg/m^2^) in early childhood is associated with obesity into later childhood [6]. Atopic sensitization and atopic disease commonly start in early childhood [7], and associations between accelerated weight gain in early childhood and subsequent atopic sensitization, allergic rhinitis [8] and asthma [9], have been suggested, but not for atopic dermatitis. Physical activity during childhood may also be associated with atopy, either directly or due to an influence on body composition [10].

Many of these associations of childhood obesity and physical activity with atopy appear in cross-sectional studies [5, 10, 11]. Few longitudinal studies exist, mainly from registers [4, 5, 11].

It is unknown if an accelerated weight gain from birth is associated with an increased risk of atopy. Furthermore, it is unknown if a positive association between BMI and asthma in children is limited to those with atopy.

The present cohort study was derived from a case control study nested within three birth cohorts that focused on preeclampsia, that had repeated anthropometric measurements, linked information across childhood, and measures of atopic disease at clinical follow-ups. The aim was to study whether weight-related anthropometrics, changes in BMI SDS and physical activity at different ages in childhood are associated with atopic sensitization and atopic disease by late childhood. We hypothesized that childhood excessive weight/obesity or accelerated weight gain is positively associated and physical activity negatively associated with atopic sensitization and atopic diseases.

## Methods

### Study population and design

The study was a part of “the Stavanger study” described in detail previously [12]. From a population-based cohort, a nested case–control study was conducted, where offspring exposed to maternal preeclampsia and unexposed offspring were identified from all births delivered in Stavanger University hospital in 1993–1995. For each exposed offspring, two matched unexposed offspring were selected: one as the next born in the hospital (i.e. a birth match) and one as the next born matched on maternal age (i.e. a risk factor for preeclampsia). 1025 children, 366 in the preeclampsia and 659 in the control group, were invited to participate in a first follow-up study at 10.8 years (girls) and 11.8 years (boys), and a second follow-up at 12.8 years (both sexes) [13]. The ages at follow-up were selected to coincide with the ages of pubertal onset and menarche of the children. Our analyses disregarded the matched pairs due to missing participants. Therefore, in our study, data were analysed as a historical cohort adjusting for preeclampsia and maternal age, including all the children who participated in both follow-ups, with predictors as listed.

The study was approved by the Norwegian Data Inspectorate, the Regional Committee for Ethics in medical research Western Norway, and the Institutional Review Boards of the National Cancer Institute and University of Texas at Austin, United States. Mothers and children signed an informed consent/assent form at follow-up.

### Outcomes

The outcomes allergic rhinoconjunctivitis, atopic dermatitis, asthma ever (evaluated at first follow-up), atopic sensitization and current asthma (evaluated at second follow-up) were defined as follows:

#### *Atopic sensitization*

**Fig. 1:**
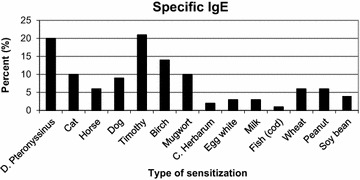
Percent of children with sensitization (specific IgE > 0.35 kU/l) to common allergens, measured at 12.8 years (n = 388). *D: dermatophagoides*, *C: cladosporium*

Blood specific immunoglobulin E (IgE) ≥ 0.35 kU/l for at least one common allergen. Blood was drawn at second follow-up and analysed by Phadiatop^®^, fx5E^®^ and by specific IgE when positive [13]. Included allergens are shown in Fig. 1. The levels of specific IgE ≥ 0.35 were added, and high grade sensitization was defined as a sum >3.9 kU/l: above the lower quartile of all children being sensitized. The ordinal outcome variable atopic sensitization was categorized as: no, low grade and high grade sensitization.

#### *Atopic disease: Asthma ever, allergic rhinoconjunctivitis or atopic dermatitis*

At first follow-up, questions on atopic disease of the child were asked to the mothers: “Has your child ever had doctor-diagnosed asthma or ever diagnosed with allergy in nose/eyes (hay fever) or atopic dermatitis (childhood eczema)?” Response of “yes” was classified as having the respective diagnosis.

#### *Current asthma*

At second follow-up, the children answered a questionnaire on reported asthma symptoms and medication during the last year according to the International Study of Asthma and Allergies in Childhood (ISAAC) and asthma ever was recorded [14]. Current asthma at second follow-up was defined as asthma ever, in addition to asthma symptoms or the use of asthma medication the last 12 months (Additional file 1).

### Predictors

Birthweight and gestational age were abstracted from hospital records. Recordings of length/height and weight measurements were collected from well-baby clinics at the target ages of 3, 6 and 12 months and 4 years. Trained nurse researchers measured height, weight, triceps skinfold and waist circumference twice in offspring at both follow-ups, and subscapular skinfold was measured twice at second follow-up; the average of each was used in the analysis [12]. Skinfolds were measured with Harpenden Skinfold Calliper^®^.

Change in weight and BMI SDS was calculated as the difference between weight and BMI SDS from each target age to the next.

At the first follow-up, the “Stanford Brief Activity Survey”, a questionnaire validated for adults, was administered to the mothers requesting responses about physical activity of the child. Specifically the answers to the following questions were extracted: “How active was your child at 3–6 years?” and “How active was your child at 6–10 years?” The response categories were categorized as: passive and/or not so active = low activity, active = normal, very active = high activity [15].

### Confounders

Potential confounders included categorical and continuous variables, i.e. sex; gestational age; birth order (firstborn or not); duration of breastfeeding (categories: none; <3; >3 months. This information was extracted from maternal questionnaire, and used in analyses for predictors at target ages ≥6 months); mother’s preeclampsia (none, mild/moderate, severe) [16]; mother’s BMI (weight at first antenatal visit and height at first follow-up); mother’s smoking (at first antenatal visit); mother’s doctor-diagnosed asthma [13]; mother’s education (from maternal questionnaire: <9; 9–12; >12 years) and mother’s age. This is illustrated in a Directed Acyclic Graph (Additional file 1: Figure S1) [17].

### Statistical methods

Descriptive statistics were analysed as means, standard deviations, numbers and percentages for the main predictors and outcomes.

There was a wide range of gestational ages at birth due to the inclusion of offspring of preeclampsia and normotensives and a wide age range at later well-child visits, analysing actual values for anthropometrics as predictors would therefore not be appropriate. Instead, standard deviation scores (SDS) based on anthropometric values and actual ages were computed from validated references [18–21]. Conversions into SDS were done using R version 2.6.2 (R Development Core Team, Vienna, Austria).

The associations between anthropometrics and physical activity for each target age (see predictors), and outcomes of atopic sensitization and atopic diseases in late childhood were analysed using multiple binary and ordinal logistic regression analyses (the latter for outcome of atopic sensitization). Separate analyses of the predictors were done for each follow-up time.

Each variable was entered separately into simple regression models. Next, all potential confounders were included in fully adjusted models. Backward stepwise selections were performed to remove non-significant confounders, unless there was ≥15 % change in effect size upon removal of the confounder. Last, final models for each target age included anthropometrics, physical activity, sex of the child and all remaining confounders.

For each predictor, odds ratios with p-values from likelihood ratio tests and 95 % confidence intervals are reported. Interactions between anthropometrics and physical activity with potential confounders were tested. The significance level was 0.05 for all tests.

Also, to study possible non-straight-line associations between BMI SDS and the outcomes, multiple fractional polynomial regression (MFPR) was used with the conservative requirement of p ≤ 0.01 for non-straight-line terms.

Due to missing values the number of participants varied between the different analyses.

IBM SPSS for windows (version 22.0.0, Chicago, Ill., USA) was used for descriptive statistics and logistic regression, and Stata SE 14 for MFPR.

## Results

### Characteristics of the participants

The numbers of participants in each follow-up have been published previously [13]. Briefly, 617 children assented to participate at the first follow-up and 470 at the second follow-up. There were more girls with high BMI in the first than second follow-up, and more children with atopic dermatitis in the first than the second follow-up. Otherwise, baseline characteristics were similar between those who assented to the first follow-up only and those who assented to both follow-ups [13]. The percentages of children sensitized to different allergens appear in Fig. 1. Clinical characteristics of the participants appear in Table 1.

**Table 1:** Descriptive statistics for predictors and outcomes for 617^a^ children included in a matched cohort study with follow-up from birth to 12–13 years in the Stavanger area, Norway

Predictors	n	Mean	SD
Birthweight (kg)	606	3.37	0.71
BMI 3 months	541	16.4	1.52
BMI 6 months	559	17.2	1.53
BMI 1 year	559	17.2	1.49
BMI 4 years	477	15.9	1.43
BMI first follow-up^b^	610	18.0	2.91
BMI second follow-up^c^	466	18.8	3.00
Waist circumference (cm) first follow-up	610	63.5	7.80
Waist circumference (cm) second follow-up	466	68.0	7.72
Triceps skinfold (mm) first follow-up	605	11.7	4.63
Triceps skinfold (mm) second follow-up	465	12.1	4.81
Waist-to-height ratio first follow-up	610	0.42	0.04
Waist-to-height ratio second follow-up	466	0.43	0.05
Subscapular skinfold (mm) second follow-up	450	8.21	3.24

	n	%
Physical activity 3-6 years	601	
Low	72	12
Normal	370	62
High	159	26
Physical activity 6–10 years	596	
Low	99	17
Normal	376	63
High	121	20
*Outcome*		
Atopic sensitization at second follow-up^d^	133/388	34
Allergic rhinoconjunctivitis ever by first follow-up	131/595	22
Atopic dermatitis ever by first follow-up	149/596	25
Asthma ever by first follow-up	53/590	9
Current asthma at second follow-up	37/458	8

*SD* standard deviation, *BMI* body mass index (kg/m^2^)

^a^Due to missing values and variation in response, the number of participants varied between the different predictors and outcomes

^b^First follow-up: 10.8 years (girls), 11.8 years (boys)

^c^Second follow-up: 12.8 years

^d^99 (25.5 %) had high grade atopic sensitization (sum of specific IgE ≥ 3.9 kU/l)

### Impact of anthropometrics (Tables 2, 3, 4)

BMI SDS at 1 year was positively associated with atopic sensitization at 12.8 years with a borderline significance (Table 2). Change in BMI SDS from 1 to 4 years and BMI SDS at 4 years were positively associated with atopic dermatitis ever at the first follow-up (Tables 2, 3).

**Table 2:** The adjusted odds ratios of atopic sensitization and atopic disease in adolescence in 617 Norwegian children by weight/BMI SDS and physical activity after backward stepwise selection of potential confounders (one model for each predictor variable)

Predictor	Outcome variable (final analyses)											
	Atopic sensitization^a,b^				Allergic rhinoconjunctivitis^a^				Atopic dermatitis^c^			
Age	n	OR	95 % CI	LR-p	n	OR	95 % CI	LR-p	n	OR	95 % CI	LR-p
*Weight SDS*												
Birth	380	1.05	(0.87, 1.26)	0.609	580	0.92	(0.77, 1.10)	0.338	544	1.04	(0.88, 1.22)	0.638
*BMI SDS*												
3 months	353	1.13	(0.90, 1.41)	0.315	521	0.91	(0.73, 1.14)	0.427	487	1.04	(0.84, 1.30)	0.694
6 months	363	1.15	(0.93, 1.42)	0.205	537	0.91	(0.74, 1.12)	0.390	503	1.05	(0.86, 1.28)	0.654
1 year	363	*1.22*	*(1.00, 1.49)*	*0.050*	537	0.97	(0.80, 1.18)	0.795	503	1.06	(0.87, 1.28)	0.557
4 years^d^	320	1.01	(0.80, 1.27)	0.934	456	0.86	(0.69, 1.08)	0.191	427	*1.32*	*(1.06, 1.65)*	*0.012*
First follow-up^e^	358	0.95	(0.79, 1.15)	0.612	562	0.87	(0.73, 1.04)	0.126	527	1.11	(0.93, 1.33)	0.225
Second follow-up^e^	354	0.96	(0.79, 1.17)	0.680				N.A.				N.A.
*Physical activity*												
At 3–6 years^f^	320			*0.039*	456			0.157	427			0.519
Normal	192	1.00	Reference		277	1.00	Reference		259	1.00	Reference	
Low	40	*2.36*	*(1.15, 4.81)*		57	1.60	(0.83, 3.09)		57	1.30	(0.65, 2.61)	
High	88	0.90	(0.50, 1.61)		122	0.77	(0.44, 1.35)		111	1.34	(0.78, 2.30)	
At 6–10 years^g^	358			0.773	562			0.126	527			*0.033*
Normal	234	1.00	Reference		353	1.00	Reference		333	1.00	Reference	
Low	57	1.05	(0.57, 1.96)		93	1.67	(0.97, 2.87)		89	1.49	(0.87, 2.56)	
High	67	0.82	(0.45, 1.49)		116	1.15	(0.68, 1.93)		105	*1.94*	*(1.16, 3.24)*	

First follow-up: 10.8 years (girls) and 11.8 years (boys); Second follow-up: 12.8 years (both sexes)

Italics numbers indicate statistically significant results

*n* number of participants, *OR* odds ratio, *CI* confidence interval, *LR*-*p* (likelihood ratio-test p-value) refers to predictor only, *BMI* body mass index (kg/m^2^), *SDS* standard deviation score, *N.A.* not applicable

^a^Adjusted for sex, preeclampsia and gestational age

^b^n = 388. Ordinal response: none, low grade, high grade (sum of specific IgE ≥ 3.9 kU/l; above the lower quartile of all children being sensitized)

^c^Adjusted for sex, mother’s asthma and mother’s smoking

^d^Also adjusted for physical activity at 3–6 years

^e^Also adjusted for physical activity at 6–10 years

^f^Also adjusted for BMI SDS at 4 years

^g^Also adjusted for BMI SDS at the first follow-up

**Table 3:** The adjusted odds ratios of atopic sensitization and atopic disease in adolescence in 617 Norwegian children by changes in weight/BMI SDS after backward stepwise selection of potential confounders (one model for each predictor variable)

Predictor	Outcome variable (final analyses)											
	Atopic sensitization^a,b^				Allergic rhinoconjunctivitis^a^				Atopic dermatitis^c^			
Age	n	OR	95 % CI	LR-p	n	OR	95 % CI	LR-p	n	OR	95 % CI	LR-p
*Weight SDS*												
Birth to 3 months	361	1.10	(0.87, 1.38)	0.408	534	0.90	(0.74, 1.09)	0.292	500	0.98	(0.80, 1.17)	0.835
*BMI SDS*												
3–6 months	351	1.15	(0.83, 1.60)	0.413	519	0.96	(0.71, 1.30)	0.794	485	1.15	(0.85, 1.56)	0.363
6 months to 1 year	360	1.30	(0.95, 1.76)	0.100	534	1.11	(0.83, 1.48)	0.470	500	1.07	(0.81, 1.42)	0.638
1–4 years	325	0.82	(0.62, 1.09)	0.173	456	0.82	(0.63, 1.07)	0.150	427	*1.46*	*(1.11, 1.92)*	*0.005*
4 years to first follow-up^d^	315	0.99	(0.78, 1.26)	0.940	450	1.01	(0.80, 1.28)	0.917	422	0.87	(0.70, 1.09)	0.223
First to second follow-up^e^	349	0.96	(0.65, 1.43)	0.843	N.A.	N.A.	N.A.	N.A.	N.A.	N.A.	N.A.	N.A

First follow-up: 10.8 years (girls) and 11.8 years (boys); Second follow-up: 12.8 years (both sexes)

Italics numbers indicate statistically significant results

*n* number of participants, *OR* odds ratio, *CI* confidence interval, *LR*-*p* likelihood ratio-test p-value refers to predictor only, *BMI* body mass index (kg/m^2^), *SDS* standard deviation score, *N.A.* not applicable

^a^Adjusted for sex, weight/BMI SDS at start of interval, gestational age, preeclampsia (none/mild or moderate/severe)

^b^n = 388. Ordinal response: none, low grade, high grade (sum of specific IgE ≥ 3.9 kU/l; above the lower quartile of all children being sensitized)

^c^Adjusted for sex, weight/BMI SDS at start of interval, mother’s asthma and mother’s smoking

^d^Also adjusted for physical activity at 3–6 years

^e^Also adjusted for physical activity at 6–10 years

**Table 4:** The adjusted odds ratios of asthma in adolescence in 617 Norwegian children by weight/BMI SDS and physical activity after backward stepwise selection of potential confounders (one model for each predictor variable)

Predictor	Outcome variable (final analyses)							
	Asthma ever by first follow-up^a^				Current asthma at second follow-up^b^			
Age	n	OR	95 % CI	LR-p	n	OR	95 % CI	LR-p
*Weight SDS*								
Birth	580	0.93	(0.72, 1.20)	0.587	439	0.95	(0.71, 1.27)	0.716
*BMI SDS*								
3 months	520	0.88	(0.64, 1.21)	0.437	411	0.96	(0.67, 1.38)	0.835
6 months	536	1.06	(0.79, 1.41)	0.707	425	0.86	(0.61, 1.20)	0.370
1 year	536	1.00	(0.76, 1.32)	0.998	424	0.78	(0.57, 1.08)	0.127
4 years^c^	454	1.09	(0.80, 1.47)	0.596	361	0.91	(0.63, 1.32)	0.627
First follow-up^d^	558	1.02	(0.79, 1.33)	0.849	426	1.10	(0.81, 1.49)	0.561
Second follow-up^d^	N.A.	N.A.	N.A.	N.A.	421	1.25	(0.90, 1.72)	0.176
*Physical activity*								
At 3–6 years^e^	454			*0.014*	361			0.475
Normal	275	1.00	Reference		220	1.00	Reference	
Low	57	*3.61*	*(1.56, 8.36)*		48	1.92	(0.66, 5.59)	
High	122	1.34	(0.61, 2.97)		93	1.40	(0.55, 3.55)	
At 6–10 years^f^	558			*0.038*	426			0.177
Normal	351	1.00	Reference		274	1.00	Reference	
Low	92	*2.52*	*(1.24, 5.12)*		69	1.98	(0.80, 4.85)	
High	115	1.02	(0.46, 2.28)		83	1.97	(0.83, 4.67)	

First follow-up: 10.8 years (girls) and 11.8 years (boys); Second follow-up: 12.8 years (both sexes)

Italics numbers indicate statistically significant results

*n* number of participants, *OR* Odds ratio, *CI* confidence interval, *LR*-*p* likelihood ratio-test p-value refers to predictor only, *BMI* body mass index (kg/m^2^), *SDS* standard deviation score, *N.A.* not applicable

^a^After stepwise backward selection, adjusted for sex, gestational age, mother’s preeclampsia and asthma

^b^After stepwise backward selection, adjusted for sex, mother’s preeclampsia and mother’s asthma

^c^Also adjusted for physical activity at 3–6 years

^d^Also adjusted for physical activity at 6–10 years

^e^Also adjusted for BMI SDS at 4 years

^f^Also adjusted for BMISDS at the first follow-up

Birthweight/BMI SDS and changes in weight or BMI SDS were not associated with allergic rhinoconjunctivitis (Tables 2, 3) or asthma (Table 4 and Additional file 2: Table S1). Skinfolds, waist circumference and waist-to-height ratio at the follow-ups were not associated with atopic sensitization or atopic disease (Additional file 2: Table S2).

### Impact of physical activity (Tables 2, 4)

Low physical activity at 3–6 years was positively associated with atopic sensitization at 12.8 years (Table 2). High physical activity at 6–10 years was positively associated with atopic dermatitis ever at the first follow-up (Table 2). Low physical activity at 3–6 and 6–10 years were positively associated with asthma ever at the first follow-up (Table 4). Physical activity was not associated with allergic rhinoconjunctivitis.

In the MFPR analyses, no non-straight-line associations were found for anthropometrics or physical activity with atopic sensitization or atopic diseases (data not shown).

## Discussion

In this cohort study of children followed from birth to 12.8 years, after adjusting for potential confounders, BMI in childhood was positively associated with atopic sensitization and atopic dermatitis in late childhood. High and low physical activity during childhood was differentially associated with atopic sensitization, atopic dermatitis and asthma assessed in late childhood. BMI and physical activity were not associated with allergic rhinoconjunctivitis.

### Anthropometrics and outcomes

In this cohort BMI at 1 year was positively associated with atopic sensitization at 12.8 years with borderline significance. As recently reviewed by Boulet [5], some studies are consistent with and others conflict our results, which may be due to differences in the study design. Studies showing an association have been cross-sectional and therefore unable to assess whether obesity precedes sensitization [5]. In our study, the majority of children were sensitized to airborne allergens. Sensitization to airborne allergens is uncommon in Scandinavian children before the age of 1 year [22]. It is therefore probable that the high BMI at 1 year preceded airborne sensitization. However, sensitization to food allergens may have been present in the first year of life, which we did not assess. In mice, obesity lowered the threshold for atopic sensitization, suggesting that obesity causes atopy [23].

Several mechanisms explaining the association between obesity and atopic sensitization have been suggested [5]. In addition to hormonal and genetic factors, a high BMI is associated with higher body fat and altered adipokines, and in turn might predispose to atopic sensitization through inflammatory changes [24]. Our results could indicate that a high BMI at the age of 1 year is of importance.

In this cohort, change in BMI SDS from 1 to 4 years and BMI at 4 years were positively associated with atopic dermatitis ever at first follow-up. In the ISAAC 3 study, overweight and obesity at 13–14 years was associated with current atopic dermatitis [10]. In a meta-analysis, mainly of cross-sectional studies, a high BMI in childhood, adolescence and adulthood was also associated with atopic dermatitis [25].

Atopic dermatitis usually has its debut in the first years of life and normally precedes any potential overweight or obesity [7]. Therefore, there may be reverse causality, but it may also be possible that overweight/obesity causes atopic dermatitis. Firstly, obesity is associated with an increased risk of dry skin, aggravating underlying skin defects [26]. Secondly, the positive association of a change in BMI SDS and overweight in preschool years with atopic dermatitis could be explained by immunological changes due to increased body fat, and an association between adipokines and atopic dermatitis has been reported [27].

Anthropometric measures were not associated with allergic rhinoconjunctivitis in accordance with previous studies [28].

In the present study, there was no association between anthropometric measures during childhood and asthma in late childhood, without variation by sex. In 2013, six studies were included in a meta-analysis showing that overweight and obesity in childhood is associated with subsequent asthma. However, the results were inconsistent regarding sex [4]. Recently, in a study including >24,000 children, accelerated weight gain from birth to 3 years was positively associated with asthma by 3 years with risk ratio of 1.22 and at 7 years with risk ratio of 1.13 [9]. Our results do not contradict this, as we may have too few participants to show a significant association of a similar low magnitude.

### Physical activity and outcomes

In this cohort, low preschool activity level was positively associated with atopic sensitization at 12.8 years. To our knowledge, this is the first study to show such an association. In a cross-sectional study of 2000 Spanish adolescents using questionnaires, there was no association between physical activity and allergy at 13–17 years [29].

In our study we adjusted for BMI, but BMI underestimates the relative amount of fat tissue in the body composition of children [30]. Thus, the association between low activity and atopic sensitization might be due to a higher body fat percentage in children with a low activity level, independent of weight status, with subsequent changes in adipokines that may influence the development of sensitization [24].

In this cohort, a high level of physical activity at 6–10 years was associated with atopic dermatitis ever at first follow-up. This is in accordance with the findings in ISAAC 3, where vigorous physical activity in children 13–14 years was associated with current atopic dermatitis, and was attributed to sweat-induced itch [10]. One possible explanation for an association is that after long-term physical activity, natural killer cell cytotoxicity could be increased, which in turn has been associated with atopic dermatitis [31, 32].

We report an association of low physical activity at both 3–6 and 6–10 years with asthma ever, but not current asthma, in late childhood. In the ISAAC 3 study, several hours of TV viewing was associated with symptoms of current asthma in adolescents [10]. Similarly, studies indicate that physical activity could be protective against the development of asthma [11]. We found no association between physical activity and allergic rhinoconjunctivitis. In ISAAC 3, both associations of vigorous physical activity and a sedentary lifestyle at 13 years with allergic rhinoconjunctivitis were found with odds ratios at 1.25 and 1.17, respectively [10]. With the low number of participants in our study, our results do not contradict these results.

### Strengths and limitations

Strengths: The study population is homogeneous regarding socio-economic status and ethnicity. Further, the predictor variables have been sampled by repeated measurements from several ages to examine the window of time in childhood the predictors could possibly affect the development of atopic sensitization and atopic diseases.

However, the study also has some limitations. The present study was not primarily designed to answer the current research questions, but this has been accounted for by including the design variable preeclampsia as a potential confounder, and no confounding was present. BMI may have limited correlation with childhood adiposity [3], and we did not have other adiposity measurements available from the visits at the well-baby clinics. Children who participated in the first follow-up but not in the second follow-up had a higher BMI and more atopic dermatitis. This may have biased our results, as both overweight and atopic dermatitis may be associated with atopic sensitization and other atopic disease. Only the predictors and not the outcomes were measured longitudinally, thus we cannot know if the associations reveal causality, or the direction of the causality. Lastly, due to several statistical analyses, the statistically significant results must be interpreted with care.

## Conclusions

The results of this study suggest that BMI and physical activity in early childhood are associated with atopic sensitization, atopic dermatitis and asthma in later childhood. Larger cohorts with repeated measurements of both predictors and outcomes are needed to further elucidate this issue.

## Additional files

10.1186/s13601-016-0124-9 Directed Acyclic Graph. Colours of rings: Green = predictor; blue with black dot = outcome; blue = ancestor of outcome; red = potential confounder; black = adjustment set; grey = unavailable/unknown confounders. Red line = biasing path; green line = causal path; black line = closed path. The figure was made by using DAGitty software.
10.1186/s13601-016-0124-9 The adjusted odds ratios of asthma in adolescence in 617 Norwegian children by changes in weight/BMI SDS after backward stepwise selection of potential confounders (one model for each predictor variable). **Table S2**. The adjusted odds ratios of atopy in adolescence in 617 Norwegian children according to weight-related anthropometry in adolescence after backward stepwise selection of potential confounders (one model for each predictor variable).

## Abbreviations

BMI: body mass index (kg/m^2^)
IBM SPSS: International Business Machines Corporation Statistical Package for the Social Sciences
IgE: immunoglobulin E
ISAAC: International Study of Asthma and Allergies in Childhood
kU/l: kilo units per litre
MFPR: multiple fractional polynomial regression
SDS: standard deviation score
