# Supplementary figures and images for: Body mass index and physical activity in early childhood are associated with atopic sensitization, atopic dermatitis and asthma in later childhood
Source: Clin Transl Allergy. 2016 Aug 24;6(1):33. doi: 10.1186/s13601-016-0124-9 (PMC4995660; doi:10.1186/s13601-016-0124-9)

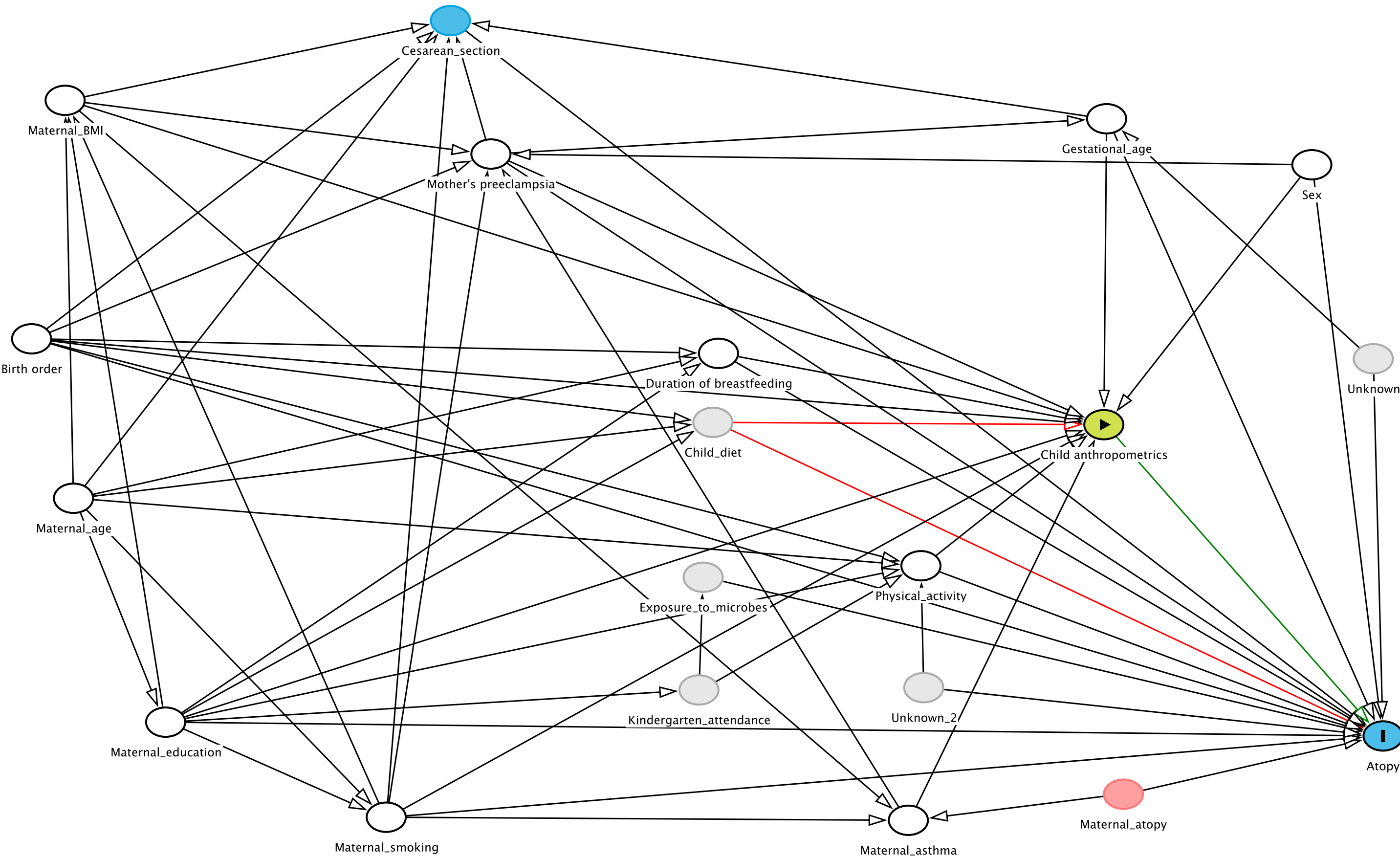

Supplement: Supplementary file 1 — 10.1186/s13601-016-0124-9 Directed Acyclic Graph. Colours of rings: Green = predictor; blue with black dot = outcome; blue = ancestor of outcome; red = potential confounder; black = adjustment set; grey = unavailable/unknown confounders. Red line = biasing path; green line = causal path; black line = closed path. The figure was made by using DAGitty software. [file 13601_2016_124_MOESM1_ESM.pdf]
