# Supplementary material for: Body mass index and physical activity in early childhood are associated with atopic sensitization, atopic dermatitis and asthma in later childhood
Source: Clin Transl Allergy. 2016 Aug 24;6(1):33. doi: 10.1186/s13601-016-0124-9 (PMC4995660; doi:10.1186/s13601-016-0124-9)
Supplement: Supplementary file 2 — 10.1186/s13601-016-0124-9 The adjusted odds ratios of asthma in adolescence in 617 Norwegian children by changes in weight/BMI SDS after backward stepwise selection of potential confounders (one model for each predictor variable). Table S2. The adjusted odds ratios of atopy in adolescence in 617 Norwegian children according to weight-related anthropometry in adolescence after backward stepwise selection of potential confounders (one model for each predictor variable). [file 13601_2016_124_MOESM2_ESM.docx]

**Table S1** The adjusted odds ratios of asthma in adolescence in 617 Norwegian children by changes in weight/BMI SDS after backward stepwise selection of potential confounders (one model for each predictor variable)

| **Outcome variable (final analyses)^a)^** | | | | | | | | |
| --- | --- | --- | --- | --- | --- | --- | --- | --- |
| **Predictor** | **Asthma ever by first follow-up** | | | | **Current asthma at second follow-up** | | | |
| **Age** | **n** | **OR** | **95% CI** | **LR-p** | **n** | **OR** | **95% CI** | **LR-p** |
| ***Weight SDS*** |  |  |  |  |  |  |  |  |
| Birth – 3 months | 533 | 0.89 | (0.68, 1.18) | 0.453 | 422 | 0.90 | (0.67, 1.20) | 0.492 |
| ***BMI SDS*** |  |  |  |  |  |  |  |  |
| 3 – 6 months | 518 | 1.42 | (0.92, 2.19) | 0.110 | 410 | 0.78 | (0.45, 1.33) | 0.358 |
| 6 – 12 months | 533 | 0.92 | (0.60, 1.39) | 0.680 | 422 | 0.77 | (0.46, 1.28) | 0.310 |
| 1 – 4 years | 454 | 1.02 | (0.69, 1.50) | 0.936 | 376 | 1.02 | (0.64, 1.60) | 0.947 |
| 4 years to First follow-up^b)^ | 448 | 0.96 | (0.68, 1.36) | 0.827 | 375 | 1.19 | (0.81, 1.76) | 0.380 |
| First to second follow-up ^c)^ |  |  |  | N.A. | 422 | 1.83 | (1.02, 3.28) | 0.054 |

*Abbreviations:* n = number of participants; OR = Odds ratio; CI = Confidence interval; LR-p = (likelihood ratio-test p-value) refers to predictor only; First follow-up: 10.8 years (girls) and 11.8 years (boys); Second follow-up: 12.8 years (both sexes); BMI = body mass index (kg/m^2^); SDS = standard deviation score; N.A. = not applicable. a) After stepwise backward selection, adjusted for weight/BMI SDS at start of interval, sex, gestational age, preeclampsia, mother’s age and mother’s asthma; b) Also adjusted for physical activity at 3-6 years; c) Also adjusted for physical activity at 6-10 years

**Table S2** The adjusted odds ratios of atopy in adolescence in 617 Norwegian children according to weight-related anthropometry in adolescence after backward stepwise selection of potential confounders (one model for each predictor variable)

| **Outcome variable (final analyses)^a)^** | | | | | | | | | | | | | | | | | | | | |
| --- | --- | --- | --- | --- | --- | --- | --- | --- | --- | --- | --- | --- | --- | --- | --- | --- | --- | --- | --- | --- |
|  | **Atopic sensitization^a)^** | | | | **Allergic rhinoconjunctivitis^a)^** | | | | **Atopic dermatitis^b)^** | | | | **Asthma ever by first**  **follow-up^c)^** | | | | **Current asthma at second**  **follow-up^d)^** | | | |
| **Predictor** | **n** | **OR** | **95% CI** | **LR-p** | **n** | **OR** | **95% CI** | **LR-p** | **n** | **OR** | **95% CI** | **LR-p** | **n** | **OR** | **95% CI** | **LR-p** | **n** | **OR** | **95% CI** | **LR-p** |
| **Waist circumference** |  |  |  |  |  |  |  |  |  |  |  |  |  |  |  |  |  |  |  |  |
| First follow-up | 367 | 0.99 | (0.81, 1.20) | 0.888 | 571 | 0.88 | (0.73, 1.06) | 0.183 | 536 | 0.97 | (0.81, 1.16) | 0.713 | 581 | 1.05 | (0.81, 1.36) | 0.699 | 445 | 1.04 | (0.76, 1.42) | 0.810 |
| Second follow-up | 368 | 0.98 | (0.79, 1.22) | 0.853 |  |  |  | N.A. |  |  |  | N.A. |  |  |  | N.A. | 447 | 1.15 | (0.81, 1.63) | 0.438 |
| **Waist-to-height ratio** |  |  |  |  |  |  |  |  |  |  |  |  |  |  |  |  |  |  |  |  |
| First follow-up | 367 | 1.03 | (0.85, 1.24) | 0.755 | 571 | 0.94 | (0.78, 1.13) | 0.508 | 536 | 1.05 | (0.88, 1.26) | 0.569 | 581 | 1.11 | (0.85, 1.44) | 0.439 | 445 | 1.08 | (0.80, 1.47) | 0.597 |
| Second follow-up | 368 | 1.04 | (0.84, 1.29) | 0.727 |  |  |  | N.A. |  |  |  | N.A. |  |  |  | N.A. | 447 | 1.15 | (0.81, 1.63) | 0.438 |
| **Triceps skinfold** |  |  |  |  |  |  |  |  |  |  |  |  |  |  |  |  |  |  |  |  |
| First follow-up | 364 | 1.03 | (0.84, 1.25) | 0.795 | 567 | 0.90 | (0.75, 1.08) | 0.253 | 532 | 1.10 | (0.92, 1.32) | 0.316 | 576 | 1.11 | (0.85, 1.44) | 0.453 | 442 | 1.20 | (0.87, 1.65) | 0.257 |
| Second follow-up | 367 | 1.02 | (0.83, 1.26) | 0.813 |  |  |  | N.A. |  |  |  | N.A. |  |  |  | N.A. | 446 | 1.23 | (0.89, 1.71) | 0.205 |
| **Subscapular skinfold** |  |  |  |  |  |  |  |  |  |  |  |  |  |  |  |  |  |  |  |  |
| Second follow-up | 358 | 0.98 | (0.79, 1.22) | 0.862 |  |  |  | N.A. |  |  |  | N.A. |  |  |  | N.A. | 447 | 1.15 | (0.81, 1.63) | 0.438 |

*Abbreviations:* n = number of participants; OR = Odds ratio; CI = Confidence interval; LR-p = (likelihood ratio-test p-value) refers to predictor only; First follow-up: 10.8 years (girls) and 11.8 years (boys); Second follow-up: 12.8 years (both sexes); BMI = body mass index (kg/m^2^); SDS = standard deviation score; N.A. = not applicable. a) After stepwise backward selection, adjusted for sex, gestational age and preeclampsia; b) After stepwise backward selection, adjusted for sex, mother’s smoking and mother’s asthma; c) After stepwise backward selection, adjusted for sex and mother’s asthma; d) After stepwise backward selection, adjusted for sex
